# Identification of Novel Zoonotic Activity of Bartonella spp., France 

**DOI:** 10.3201/eid2203.150269

**Published:** 2016-03

**Authors:** Muriel Vayssier-Taussat, Sara Moutailler, Françoise Féménia, Philippe Raymond, Olivier Croce, Bernard La Scola, Pierre-Edouard Fournier, Didier Raoult

**Affiliations:** Agence Nationale de Sécurité Sanitaire de l’Alimentation, de l’Environnement et du Travail, Maisons Alfort, France (M. Vayssier-Taussat, S. Moutailler, F. Féménia);; Groupe Chronimed, Saint-Péray, France (P. Raymond);; Aix-Marseille Université, Marseille, France (O. Croce, B. La Scola, P-E. Fournier, D. Raoult)

**Keywords:** Bartonella, zoonoses, tickborne infections, ticks, bacteria, vector, vector-borne infections, bacteremia, France

## Abstract

Certain *Bartonella* species are known to cause afebrile bacteremia in humans and other mammals, including *B. quintana*, the agent of trench fever, and *B. henselae*, the agent of cat scratch disease. Reports have indicated that animal-associated *Bartonella* species may cause paucisymptomatic bacteremia and endocarditis in humans. We identified potentially zoonotic strains from 6 *Bartonella* species in samples from patients who had chronic, subjective symptoms and who reported tick bites. Three strains were *B. henselae* and 3 were from other animal-associated *Bartonella* spp. (*B. doshiae, B. schoenbuchensis*, and *B. tribocorum*). Genomic analysis of the isolated strains revealed differences from previously sequenced *Bartonella* strains. Our investigation identifed 3 novel *Bartonella* spp. strains with human pathogenic potential and showed that *Bartonella* spp. may be the cause of undifferentiated chronic illness in humans who have been bitten by ticks.

## Abstract 2

These bacteria may cause paucisymptomatic bacteremia and endocarditis in humans.

*Bartonella* spp. cause varied and multifaceted human diseases, including cat scratch disease (*B. henselae*), Carrion’s disease (*B. bacilliformis*), trench fever (*B. quintana*), endocarditis (*B. quintana* and *B. henselae*) (*1*,*2*), bacillary angiomatosis (*B. quintana* and *B. henselae*), and hepatic peliosis (*B. henselae*). *Bartonella* spp. can also cause prolonged intra-erythrocytic bacteremia in both humans and animals (*3*): in humans, *B. quintana*, *B. bacilliformis*, and *B. rochalimae* are known pathogens, and in animals, *B. henselae*, *B. clarridgeiae*, and *B. koehlerae* have been identified in felids; *B. grahamii, B. taylorii, B. doshiae, B. birtlesii*, and others in rodents; and *B. bovis, B. chomelii, B. schoenbuchensis,* in ruminants. In humans, chronic bacteremia caused by *B. quintana* causes few obvious symptoms apart from generalized fatigue and nonspecific leg pain (*1*,*4*).

It has been assumed that each *Bartonella* species infected 1 or a few closely related mammalian reservoir hosts, in which infection caused long-lasting bacteremia. Nonreservoir hosts were considered incidentally infected without bacteria being detected in blood. Recently, these assumptions has been contradicted by studies describing animal-associated *Bartonella* spp. indirectly associated with bacteremia and a spectrum of diverse symptoms in immune-competent persons who had contact with animals, arthropods, or both, which are natural routes of *Bartonella* transmission (*5*–*7*). In some cases, the source of infection remains unknown; ticks have been suggested as a possible source of animal-associated *Bartonella* infection in humans (*6*,*8*–*10*).

Related to a patient’s history of tick bites, it is common for physicians to suspect Lyme disease, some rickettsial diseases, or tickborne encephalitis. However, in many cases, the diagnosis is not confirmed by serologic or DNA-based tests. In recent years, alternate interpretations of Lyme disease serology have flourished, leading to considerable discord between formal institutions for infectious disease and patient advocacy associations. Thus, unexplained symptoms after tick bites have become an issue of increasing importance for patients and their physicians (*11*,*12*).

In this context, we screened for the presence of *Bartonella* in the blood of patients reporting tick bites and with unexplained and aspecific symptoms. Here we report the isolation and genomic sequencing of 6 *Bartonella* strains obtained by blood culture from 66 patients. Three strains were identified as *B. henselae*, and 3 other strains were identified as different animal–associated species (*B. doshiae, B. tribocorum*, and *B. schoenbuchensis*)*.*

## Methods

### Patients

During January–June 2013, we conducted a study of a cohort of 66 French patients who had consulted their doctors for chronic symptoms appearing after a tick bite. The entire study protocol was approved by the ethics committee of the Institut Federatif de Recherche 48 under reference 13–022–1.

All patients associated symptom onset with tick bites that occurred during 2008–2012 (Table 1). At symptom onset, local doctors were consulted, and serologic tests for Lyme borreliosis were performed. All patient samples tested were seronegative for Lyme borreliosis bacteria; however, since that time, their symptoms had become chronic. The patients completed information forms giving informed consent for the use of their samples in the study. All of the patients lived in the countryside, where ticks were abundant and contact with wild animals was possible. The patients reported that they had not undergone antibacterial drug treatment for >3 months before the study. 

**Table 1:** Patients whose blood cultures were positive for *Bartonella* spp. that had no previously known zoonotic activity, France

Case-patient no./age, y/sex	*Bartonella* spp.	Tick bite date	Pets	Wild animal contact	Main complaints	Bacteremia, CFU/mL
1/49/F	*B. henselae*	Multiple since 2008	Cats, dogs, horses	Rats, fish	Fatigue, muscle pain, headache	50
2/58/M	*B. henselae*	2011	Birds, rabbits	No	Fatigue, muscle pain	70
3/47/F	*B. henselae*	2012	Dog, hamster	No	Fatigue, generalized pain, insomnia	80
4/45/F	*B. doshiae*	2009	No	No	Fatigue, blurred vision, arthralgia	50
5/64/M	*B. tribocorum*	2012	Dog	Game animals (hunter)	Fatigue, muscle pain, headache	60
6/40/F	*B. schoenbuchensis*	2011	No	No	Fatigue, muscle pain, fever	850

We collected blood samples from each patient in EDTA-containing sample tubes. For a control population, we used anticoagulated blood samples from 70 anonymous healthy blood donors from Paris (France). All samples (control and patients) were tested simultaneously. 

### *Bartonella* Isolation from Blood

To specifically isolate *Bartonella* spp., 100 μL of blood samples from patients or healthy donors were directly plated onto sheep blood agar plates and incubated at 35°C in a humidified atmosphere with 5% CO_2_ for 45 days. The plates were assessed daily from days 7–45 before the culture was deemed negative (i.e., absence of colony in the absence of contamination) (*1*). Colony-forming units (CFU) were counted and bacteremia (UFD/mL of blood) evaluated.

### Genome Sequencing, Assembly, and Analysis

We extracted genomic DNA from each isolated strain by using the EZ1 automated extraction system (QIAGEN, Hilden, Germany), following the manufacturer’s recommendations. Bacterial genomic DNA was sequenced by using the Nextera XT DNA sample prep kit (Illumina Inc., San Diego, CA, USA) and a 2×250 paired-end protocol with the MiSeq pyrosequencer (Illumina), according to the manufacturer’s instructions. We aligned each genome by using Mira version 3.2 software in the mapping mode (*13*). The resulting contigs were combined by using Opera version 1.2 (*14*) and GapFiller (*15*) software. Finally, the genomic assemblies were improved with manual refinement by using the CLC Genomics version 4.7.2 software package (CLC Bio, Aarhus, Denmark). Noncoding genes and miscellaneous features were predicted by using RNAmmer (*16*) and ARAGORN (*17*). Coding DNA sequences were predicted by using Prodigal (*18*), and functional annotation was achieved by using BLAST+ (*19*) and HMMER3 (*20*) against the UniProtKB database (*21*). Coding DNA sequences were also annotated by using the Clusters of Orthologous Groups databases (*22*) with blastp (http://blast.ncbi.nlm.nih.gov/Blast.cgi) default parameters.

Single-nucleotide polymorphisms (SNPs) among genomes were identified by using SNIT software (*23*). SNPs were searched in regions exhibiting >95% nt sequence identity and the SNIT software was used with default parameters except for the Tandem Repeat Finder filter for avoiding ambiguous SNPs in repeat regions. We also performed in silico DNA–DNA hybridization (DDH) between *Bartonella* strains by using GGDC software (*24*).

### Taxonomic Classification

To determine the taxonomic classification of the 6 isolates, we used previously proposed criteria (*25*) in which the *gltA* and *rpoB* gene sequences from each strain were compared to those of validated published *Bartonella* species. These criteria classify *Bartonella* isolates within a particular species if they share >96% and 95.4% nucleotide sequence similarity for the *gltA* and *rpoB* genes, respectively (*25*). In our study, *gltA* and *rpoB* sequences were retrieved from the genomes.

## Results

### *Bartonella* spp. Isolation

*Bartonella* spp. were isolated by prolonged culture from blood samples of 6 of the 66 patients who reported chronic symptoms following a tick bite. In contrast, samples from the 70 healthy blood donors remained negative after 45 days of incubation.

Bacteremia in the *Bartonella* infected patients increased from 50 to 850 CFU/mL. For 1 patient (case-patient 2), we had access to 2 blood samples that were taken at a 1-month interval. *B. henselae* was grown from the 2 samples, with similar bacteremia (50 and 60 CFU/mL, respectively), suggesting chronic bacteremia.

The case-patients who tested positive for *Bartonella* (Table 1) reported tick bites occurred 1–5 years before blood samples were collected. All of them live in the countryside, in contrast to the healthy blood donors, who were all from Paris, France. The main complaint of the case-patients was chronic fatigue, but they also reported other subjective or nonspecific symptoms (or both), such as headaches and myalgia. A qualifying characteristic of the 70 healthy blood donors was absence of chronic fatigue. Even though potential exposure to ticks is difficult to evaluate, because the anonymous blood donors all lived in Paris, we assumed they were not likely to have frequent tick exposure or wild animal contact.

### Taxonomic Classification

Of the 6 *Bartonella* isolates from this study, 3 (MVT01, MVT02, and MVT03) were classified within the *B. henselae* species on the basis of both their phylogenetic position and *gltA* and *rpoB* sequence similarities (Figure; Tables 1, 2). The isolates from samples from case-patient 2 at a 1-month interval shared 100% identity, based on *gltA* and *rpoB* gene comparison. Isolates MVT04, MVT05, and MVT07 were classified within the *B. tribocorum, B. doshiae,* and *B. schoenbuchensis* species, respectively.

**Figure:**
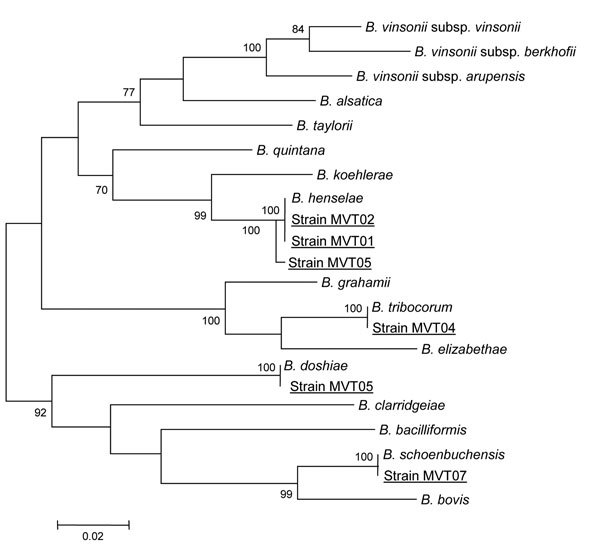
*rpoB* gene-based phylogenetic tree showing the relationships of 6 *Bartonella* isolates (underlined). Briefly, *rpoB* nucleotide sequences were aligned by using ClustalW software (http://www.clustal.org/clustal2/), and phylogenetic relationships were inferred by using the maximum-likelihood strategy and MEGA software (http://www.megasoftware.net). Bootstrap values above 70%, obtained from 500 analyses, are indicated at the nodes. Scale bar represents a 2% nucleotide sequence divergence.

**Table 2:** Nucleotide similarity of 6 *Bartonella* isolates from patients in France expressing novel zoonotic activity and their most phylogenetically similar published validated species*

Isolate	*gltA,* %	*rpoB,* %	Species
MVT01	100	100	*B. henselae*
MVT02	100	100	*B. henselae*
MVT03	99.7	99.6	*B. henselae*
MVT04	100	100	*B. tribocorum*
MVT05	98.7	100	*B. doshiae*
MVT07	100	99.9	*B. schoenbuchensis*

*gltA and *rpoB* sequences were obtained from genomic sequences. Genome sequences of *B. henselae* strains MVT01, MVT02, and MVT03 were deposited in GenBank under accession numbers HG965802, NZ_LN879429, and HG969191, respectively; the genome sequence of *B. tribocorum* strain MVT04 was deposited in GenBank under accession numbers HG969192 and HG969193; the genome sequence of *B. doshiae* strain MVT05 was deposited in GenBank under accession numbers CCBL010000001–CCBL010000013; the genome sequence of *B. schoenbuchensis* strain MVT07 was deposited in GenBank under accession numbers HG977193–HG977197; the genome sequences of the reference strains *B. henselae* strain Houston-1, *B. tribocorum* strain CIP 105476, *B. doshiae* strain NCTC 12862, and *B. schoenbuchensis* strain m07a are available in genBank under accession numbers NC_005956, NC_010161, and NC_010160, NZ_JH725094 to NZ_JH725100 and NZ_KB915627-NZ_KB915629, NZ_CM001846-NZ_CM001845, respectively.

The assembly data and main genomic characteristics of each isolated strain are summarized in Table 3; in silico DDH values and SNP numbers are described in Table 1. All studied strains displayed a similar genomic content when compared with reference genomes. The GGDC software we used proposes that DDH values >70% could classify isolates in the same species. Here, intraspecies values ranged from 80.3% to 100% (Table 4). Nevertheless, from 10 to 1,938 SNPs were identified among *B. henselae* isolates MVT01, MVT02, and MVT03 and from 693 to 2,093 SNPs when comparing these strains to *B. henselae* Houston-1 (Table 4), confirming that each strain was unique and did not result from cross-contamination or contamination from laboratory strains. 

**Table 3:** Assembly information and main characteristics of 6 sequenced *Bartonella* genomes from patients in France expressing novel zoonotic activity

Genome characteristics	Species and isolate identification					
	*B. henselae* MVT01	*B. henselae* MVT02	*B. henselae* MVT03	*B. tribocorum* MVT04	*B. doshiae* MVT05	*B. schoenbuchensis* MVT07
GenBank accession nos.	HG965802	NZ_LN879429	HG969191	HG969192–HG969193	CCBL010000001–CCBL010000013	HG977193–HG977197
Size, bp	1,902,535	1,905,383	1,975,503	2,609,404	1,919,109	1,734,324
No. contigs	1	1	1	2	13	5
Average read coverage	87	94	110	46	15	41
Average read length, trimmed	183	190	192	194	168	193
Total no. reads, trimmed	946,882	1,034,894	1,263,492	738,522	261,0852	666,371
Total no. predicted genes	1,659	1,658	1,726	2,335	1,720	1,574
Protein-coding genes	1,603	1,602	1,668	2,279	1,654	1,519
rRNA operons	2	2	2	2	2	2
tRNAs	43	43	45	43	53	41
Other RNAs	7	7	7	7	9	8
GC% content	38.18	38.18	38.09	38.84	37.82	35.58
Plasmid	0	0	0	1	0	1
Genome used as a reference for assembly (accession nos.)	*B. henselae* Houston-1 (NC_005956)	*B. henselae* Houston-1 (NC_005956)	*B. henselae* Houston-1 (NC_005956)	*B. tribocorum* CIP 105476 (NC_010161, NC_010160)	*B. doshiae* NCTC 12862 (NZ_JH725094– NZ_JH725100)	*B. schoenbuchensis* m07a (NZ_KB915627–NZ_KB915629, NZ_CM001846, NZ_CM001845)

**Table 4:** Comparisons of 6 sequenced genomes of *Bartonella* spp. isolated from humans in France expressing novel zoonotic activity showing relationships to each other and to those of closely related strains

Compared genomes (GenBank accession nos.)	*B. henselae* MVT01	*B. henselae* MVT02	*B. henselae* MVT03	*B. tribocorum* MVT04	*B. doshiae* MVT05	*B. schoenbuchensis* MVT07	*B. henselae* Houston-1	*B. tribocorum* CIP 105476	*B. doshiae* NCTC 12862	*B. schoenbuchensis* m07a
*B. henselae* MVT01 (HG965802)	*100%	100% ± 0.05	92.90% ± 1.74	28.20% ± 2.43	28.10% ± 2.43	23.80% ± 2.38	98.30% ± 0.64	28.20% ± 2.43	28.00% ± 2.43	23.60% ± 2.38
*B. henselae* MVT02 (NZ_LN879429)	10	100%	93.00% ± 1.72	28.20% ± 2.43	28.10% ± 2.43	23.80% ± 2.39	98.30% ± 0.64	28.20% ± 2.43	28.00% ± 2.43	23.60% ± 2.38
*B. henselae* MVT03 (HG969191)	1,938	1,937	100%	28.00% ± 2.43	28.00% ± 2.43	23.80% ± 2.39	92.30% ± 1.82	28.00% ± 2.43	27.90% ± 2.43	23.80% ± 2.39
*B. tribocorum* MVT04 (HG969192–HG969193)	NA	NA	NA	100%	26.50% ± 2.42	22.70% ± 2.37	28.20% ± 2.43	99.30% ± 0.33	26.60% ± 2.42	22.50% ± 2.36
*B. doshiae* MVT05 (CCBL010000001–CCBL010000013)	NA	NA	NA	NA	100%	23.60% ± 2.38	28.10% ± 2.43	26.50% ± 2.42	81.40% ± 2.72	23.60% ± 2.38
*B. schoenbuchensis* MVT07 (HG977193–HG977197)	NA	NA	NA	NA	NA	100%	23.70% ± 2.38	22.70% ± 2.37	23.60% ± 2.38	80.30% ± 2.77
*B. henselae* Houston-1 (NC_005956)	693	698	2,093	NA	NA	NA	100%	28.20% ± 2.43	28.00% ± 2.43	23.60% ± 2.38
*B. tribocorum* CIP 105476 (NC_010161, NC_010160)	NA	NA	NA	51	NA	NA	NA	100%	26.60% ± 2.42	22.60% ± 2.37
*B. doshiae* NCTC 12862 (NZ_JH725094–NZ_JH725100)	NA	NA	NA	NA	>10,000	NA	NA	NA	100%	23.60% ± 2.38
*B. schoenbuchensis* m07a (NZ_KB915627–NZ_KB915629, NZ_CM001846, NZ_CM001845)	NA	NA	NA	NA	NA	>10,000	NA	NA	NA	100%

*All values are percentage identity + SD. Isolates designated MVT01–07 and MVT07 are isolates from humans in France. Gray shading indicates in silico DNA–DNA hybridization values obtained by using Genome-to-Genome Distance Calculator software (http://ggdc.dsmz.de/); darker shading indicates values >70%. Unshaded values are SNP numbers calculated by using SNP identification for strain typing software (*23*). NA, not applicable because SNP numbers cannot be estimated for distant genomes.

Of note, *B. tribocorum* isolate MVT04 and *B. schoenbuchensis* isolate MVT07 were the only 2 that exhibited plasmids. However, when compared with reference strain m07a, MVT04 and MVT07 carried a large plasmid and not the small plasmid homologous to the cryptic pBGR plasmid harbored by *B. grahamii* (*26*).

## Discussion

In this study, animal-associated *Bartonella* isolates were individually cultured from the blood of patients who had been bitten by ticks and reported subjective symptoms, whereas no strains were isolated from healthy blood donors. This report describes the isolation of 3 different animal-associated *Bartonella* species from human samples, highlighting their potential novel zoonotic properties. Moreover, we found that zoonotic *Bartonella* spp. can be detected in the blood of afebrile patients, as has been shown for human-specific *B. quintana* and *B. bacilliformis* and as was recently reported for *Candidatus* Bartonella ancashi (*27*). Chronic bacteremia caused by infection by *Bartonella* spp. is well-described in many mammals, including humans (*4*,*28*). The *Bartonella*–mammalian host association is considered to be species-specific and attributable to co-evolution between host and pathogen (*28*). However, we show that animal-associated species can also chronically infect human blood, highlighting the possibility of host shift despite apparent host specificity (*28*,*29*).

This work is similar to that of E.B. Breitschwerdt et al. (*5*–*7*), who also recovered zoonotic *Bartonella* spp. from human samples using an in-house technique based on results of blood pre-enrichment followed by PCR detection of *Bartonella* spp.; members of the same team have investigated many cases of persons who had nonspecific symptoms, including arthralgia, muscle pain, fatigue, headaches, visual blurring, neurocognitive symptoms, and, in 2 case-patients, hemangioendothelioma (*30*). In total, *B. henselae* DNA was detected in 47 cases (*5*,*30*–*33*); *B. koehlerae* (another common agent of feline bacteremia) DNA in 96 cases, including 2 co-infected with *B. henselae* (*31*,*32*,*34*,*35*); and *B. vinsonii berkhoffii* (an agent of canine bacteremia and endocarditis) DNA in 24 cases (*31*–*34*), including 16 case-patients with *B. henselae* and 2 cases of *B. melophagi* (*36*). These results have been questioned because minute levels of contamination can result in false positives by PCR. Therefore, we deliberately avoided PCR to overcome this problem, and the resulting strain isolation was consequently straightforward and indisputable. These isolates (Table 2) have been archived in our collection (Collection de Souches de l’Unité des Rickettsies, World Data Center for Microorganisms no. 875, http://www.mediterranee-infection.com/article.php?laref=14&titre=collection-de-souches) and are available upon request under references B546, B547, B548, B549, B550, and B551 for isolates MVT01, MVT02, MVT03, MVT04, MVT05, and MVT07, respectively.

Our findings also confirm studies identifying zoonotic *Bartonella* in the blood of patients with nonspecific complaints. Among them, *B. henselae* is well known worldwide as a zoonotic agent infecting both cats and their fleas and has also been found in ticks (*10*). *B. henselae* has been detected in the blood of a patient without apparent symptoms 4 months after recovering from cat scratch disease. For this particular case, the sequence of manifestation of cat scratch disease, then bacteremia, followed by endocarditis was proposed because it has been known to occur for *B. quintana* bacteremia. One of the case-patients in this study owns a cat and may have been infected by this pet.

The 3 other animal-associated species we detected should now be considered zoonotic *Bartonella* spp. *B. doshiae* and *B. tribocorum* are both rodent-associated species; in France and worldwide, these species have mainly been recovered from rats (*Microtus agentiis* for *B. doshiae* and *Rattus rattus* for *B. tribocorum*). *B. schoenbuchensis* is normally found in deer, elk, and cattle (*37*,*38*).

The zoonotic agents we isolated from patients from France have also been detected in animals in France. Similarly, in the United States and Thailand, *Bartonella* species known to be prevalent in animals have also been identified in humans: (*B. henselae, B. vinsonii berkhoffii,* and *B. koehlerae* in the United States (*33*,*35*) and *B. tribocorum* and *B. rattimassiliensis* in Thailand (*39*). Therefore, the zoonotic *Bartonella* species discovered in humans in this study generally appear to be related to the prevalence among animals.

The significance of these *Bartonella* spp. in the genesis of the clinical picture is difficult to determine. *Bartonella* spp. are present in ticks, and we have previously reported *Bartonella* infections following tick bites, such as SENLAT (scalp eschar and neck lymphadenopathy after tick bite [*40*]). However, the causal link between the conditions observed here, *Bartonella* and tick bite, cannot yet be concretely established, especially for persons with tick bites occurring up to 5 years previously, which introduces innumerable potential confounding exposures within the same period, including bites by other arthropods. For instance, 1 of the 3 patients with *B. henselae* bacteremia reported contact with cats; this contact was a more plausible source of infection than tick bites. Furthermore, it is crucial to determine whether *Bartonella* played a notable role in the observed pathologies, because treatment for chronic *Bartonella* bacteremia (as for *B. quintana*) is particularly arduous and may require 6 weeks of doxycycline treatment together with 3 weeks of gentamicin, as these are the only antimicrobial drugs known to be effective in eradication of *Bartonella* (*1*). Many *Bartonella* spp. can also cause endocarditis, including *B. quintana* and *B. henselae*; therefore, reports of rare cases of endocarditis attributed to zoonotic *Bartonella* such as *B. kohlerae*, *B. alsatica*, *Candidatus* B. mayotimonensis*, B. vinsonii*, or *B. elizabethae* may actually be the final manifestation of asymptomatic bacteremia, similar to that reported by our infected patients (*28*).

In summary, our major finding is the isolation of zoonotic *Bartonella* other than *B. quintana* in the blood of patients with poorly qualified syndromes. These results indicate that zoonotic *Bartonella* spp. infection may cause undifferentiated chronic illness in humans.
